# Free Sugar Intake and Dementia Risk: A Swedish Cohort Study on Dietary Sources and Dementia Subtypes

**DOI:** 10.1016/j.tjnut.2026.101518

**Published:** 2026-04-01

**Authors:** Naiqi Zhang, Jenny Andresen, Suzanne Janzi, Isabelle Glans, Jessica Samuelsson, Katarina Nägga, Yan Borné, Sebastian Palmqvist, Oskar Hansson, Emily Sonestedt

**Affiliations:** 1 Nutritional Epidemiology, Department of Clinical Sciences Malmö, Lund University, Malmö, Sweden; 2 Department of Food and Meal Science, Faculty of Natural Science, Kristianstad Universirty, Kristianstad, Sweden; 3 Clinical Memory Research Unit, Department of Clinical Sciences Malmö, Lund University, Lund, Sweden; 4 Memory Clinic, Skåne University Hospital, Malmö, Sweden; 5 Neuropsychiatric Epidemiology Unit, Department of Psychiatry and Neurochemistry, Institute of Neuroscience and Physiology, Sahlgrenska Academy, Centre for Ageing and Health, University of Gothenburg, Mölndal, Sweden; 6 Department of Geriatrics and Palliative Medicine, and Department of Health, Medicine and Caring Sciences, Linköping University, Linköping, Sweden

**Keywords:** Free sugar, Sugar-sweetened beverages, Dementia, Alzheimer’s disease, Vascular dementia, *APOE* ε4

## Abstract

**Background:**

Dementia is a growing public health concern, and although diet is a modifiable potential risk factor, the role of free sugar intake remains unclear. Excess sugar has been linked to metabolic and cardiovascular dysfunction, both associated with cognitive decline, but evidence regarding specific sugar sources is limited.

**Objectives:**

This study aimed to investigate the associations between free sugar intake, its dietary sources, and the risk of all-cause dementia, Alzheimer’s disease, and vascular dementia, and to assess potential modification by apolipoprotein E (*APOE*) ε4 status.

**Methods:**

We included 27,786 participants without dementia at baseline (mean age: 58 y; 61% females) from the Malmö Diet and Cancer Study, a population-based prospective cohort. Dietary intake was assessed using a validated diet history method. Dementia diagnoses were obtained from national registers and validated by memory clinic physicians. During a median follow-up of 25 y, 3224 participants (11.6%) were diagnosed with dementia.

**Results:**

Free sugar intake was not significantly associated with all-cause dementia or Alzheimer’s disease. However, a U-shaped association was observed for vascular dementia, with moderate intake (10%–12.5% of energy) associated with lower risk [hazard ratio (HR): 0.70; 95% confidence interval (CI): 0.52, 0.95]. Sugar-sweetened beverage intake showed no association with dementia risk. High chocolate intake was associated with lower risks of all-cause [HR for quintile 5 (Q5) compared with Q1: 0.81; 95% CI: 0.72, 0.91] and vascular dementia (HR for Q5 compared with Q1: 0.68; 95% CI: 0.50, 0.92), whereas high jam/marmalade intake was linked to a lower risk of all-cause dementia (HR: 0.86; 95% CI: 0.77, 0.97 for >10 servings per week compared with <0.5 servings per week). No significant interactions with *APOE* ε4 status were observed.

**Conclusions:**

Free sugar intake was not associated with overall dementia risk, but moderate intake may reduce the risk of vascular dementia. These findings suggest that future dietary guidelines for cognitive health should consider not only sugar quantity but also its food source.

## Introduction

Dementia is a collective term for disorders characterized by progressive decline in memory and cognitive abilities. Alzheimer’s disease is the most prevalent subtype, accounting for 60%–70% of cases [1], followed by vascular dementia, which contributes to ∼15% of cases [2]. Although they have distinct features, such as neuronal loss in Alzheimer’s disease and cerebrovascular damage in vascular dementia, they often share overlapping mechanisms, including neurodegeneration, vascular injury, and impaired synaptic signaling [3,4]. Beyond its clinical impact, dementia imposes substantial burdens on individuals and healthcare systems [1]. In Sweden, around 150,000 individuals lived with dementia in 2019, a figure projected to reach 250,000 by 2050. Globally, dementia prevalence is expected to rise due to population aging [5]. However, declining rates in some high-income countries suggest that modifiable risk factors, including diet, may play a key role in prevention [6,7].

Dietary strategies such as adherence to the Mediterranean diet or Mediterranean–Dietary Approaches to Stop Hypertension Intervention for Neurodegenerative Delay diet have been associated with slower cognitive decline and lower risk of developing dementia [8]. A common feature of these diets is the low intake of foods high in added and free sugars. Free sugars, defined as those added during manufacturing or preparation, as well as sugars naturally present in honey, syrups, and fruit juices [9], are rapidly absorbed and have been linked to metabolic disturbances, insulin resistance, hyperglycemia, and cardiovascular dysfunction, all of which may contribute to increased dementia risk [[10], [11], [12], [13], [14]]. In addition, sugar-sweetened foods often lack micronutrients and dietary fiber found in foods that contain naturally occurring sugars, such as fruits and vegetables.

Several studies have suggested a possible association between high-sugar intake and dementia or cognitive decline, though findings are mixed [[15], [16], [17], [18], [19]]. For example, Zhang et al. [18] reported that both total sugar intake and a high-sugar dietary pattern were associated with increased risk of all-cause dementia in over 210,000 participants from the UK Biobank. Another study within the same cohort found a J-shaped association between free sugar intake and dementia risk [16]. Importantly, the metabolic effect of sugar may depend on its form. Liquid sugars, such as those in sugar-sweetened beverages, may exert more adverse effects than solid forms. High intake of sugar-sweetened beverages is strongly linked to weight gain, type 2 diabetes, and cardiovascular disease [9]. One hypothesis is that liquid sugar fails to trigger satiety effectively, thereby promoting excess energy intake and eventually weight gain [14]. Another mechanism focuses on the rapid absorption of fructose in these beverages, which places a high metabolic load on the liver and can drive insulin resistance [20]. In a meta-analysis of 9 cohorts and 3 cross-sectional studies, high intake of sugar-sweetened beverage consumption was significantly associated with cognitive disorders among middle-aged and older populations [19]. Schaefer et al. [16] found a linear association between free sugars from beverages and dementia risk in the UK Biobank, whereas no such link was found for solid sugars.

It remains unclear whether sugar intake influences different dementia subtypes in distinct ways, particularly vascular dementia, which has strong links to metabolic and cardiovascular dysfunction [2]. In addition, the apolipoprotein E (*APOE*) ε4 variant is the strongest common genetic risk factor for Alzheimer’s disease [21], yet its interaction with sugar intake remains unclear. Therefore, the objective of this study was to investigate the association between free sugar intake, as well as specific dietary sources of free sugar, and the risk of all-cause dementia, Alzheimer’s disease, and vascular dementia. In addition, we examined whether these associations were modified by *APOE* ε4 carrier status.

## Methods

### Study population

This study was conducted within a large Swedish cohort with long follow-up and detailed data on dietary intakes and dementia diagnoses, consisting of individuals from the Malmö Diet and Cancer (MDC) study, a population-based prospective cohort in southern Sweden. All females born between 1923 and 1950 and males born between 1923 and 1945 who were living in Malmö, Sweden, were invited to participate, yielding a source population of 74,138 individuals [22,23]. Baseline examinations were conducted between 1991 and 1996, and a total of 28,028 individuals completed dietary assessment, self-administered lifestyle questionnaire, and clinical measurements, including weight and height recorded by registered nurses. Ethical approval was obtained from the Regional Ethical Review Board in Lund, Sweden (LU/90-51), and written informed consent was obtained from all participants before participation.

### Baseline data collection

**FIGURE 1:**
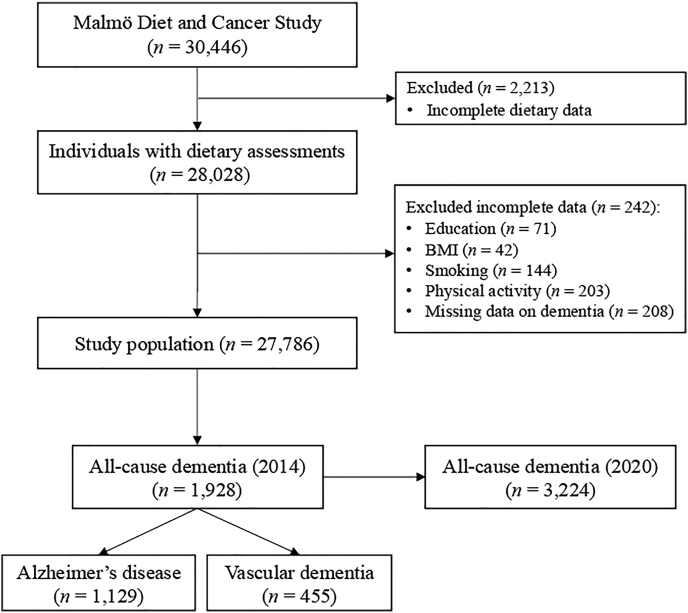
Recruitment flow and diagnosis count.

Baseline data, including information on medication use, medical history, socioeconomic status, and lifestyle factors such as leisure-time physical activity, smoking, educational level, and alcohol habits, were collected from a questionnaire. Leisure-time physical activity was assessed based on the duration of 17 different activities, and metabolic equivalent of task (MET) hours per week were calculated based on the intensity of the activities [24]. Alcohol consumption (g/d) was assessed using 7-d food diary. Diabetes was identified through self-reported diagnosis, medication usage, and registry records. Participants who reported substantial dietary changes before baseline were identified through the questionnaire [25], and potential energy misreporters were detected using the Goldberg method based on estimated energy expenditure [26]. After excluding participants with missing data on covariates such as education, BMI, smoking, and physical activity, the final analytic sample consisted of 27,786 participants (Figure 1).

### Dietary assessment

Dietary data were collected at baseline using a modified diet history method, comprising [1] a 7-d food diary (covering cooked meals, cold beverages, and dietary supplements), [2] a 168-item diet questionnaire (capturing the general meal pattern, frequency, and portion sizes of foods with low day-to-day variation—e.g., hot beverages, sandwiches, edible fats, breakfast cereals, yogurt, milk, fruits, cakes, candies, and snacks), and [3] a diet history interview. The interview, which originally lasted 60 min until 1 September, 1994, and 45 min thereafter to reduce interview time, gathered information on portion sizes and cooking methods of the food recorded in the diary. Subsequent evaluations indicated that these changes in dietary assessment routines had only minor effects on the ranking ability or mean dietary intakes [27]. Average daily nutrient and energy intakes were estimated using the MDC Food and Nutrient Database, originating from the Swedish National Food Agency. An 18-d weighed food record was used to validate a similar diet history method and demonstrated a moderately strong correlation for sucrose (Pearson’s correlation coefficients = 0.74 for females and 0.60 for males) [28].

#### Free sugar intake estimation

Free sugar intake was estimated by subtracting naturally occurring sugars from fruit and vegetables from total sucrose and monosaccharide intake, including intake from honey and syrup [29]. Free sugar intake was categorized into 5 groups based on percentage of nonalcoholic energy intake (E%) to examine a range of intakes, including extremes: <7.5 E%, >7.5–10 E%, >10–12.5 E%, >12.5–15 E%, and >15 E%.

#### Sugar-sweetened foods and beverages

Foods and beverages containing added sugars were categorized into treats (pastries, ice cream, sweets, and chocolate), toppings (table sugar, honey, jams, and marmalades), and sugar-sweetened beverages (sweetened sodas and fruit drinks, excluding 100% fruit juices) [29,30]. Treats typically contain higher fat content, whereas toppings contain negligible fat and protein. It was hypothesized that treats are more prone to overconsumption than toppings. Given evidence suggesting different metabolic effects of liquid compared with solid sugar, [31] sugar-sweetened beverages were analyzed separately.

The consumed amounts of treats, toppings, and sugar-sweetened beverages were converted from grams per day to servings per week based on standard serving sizes from the Swedish National Food Agency and manufacturers. Intake categories were defined as follows: sugar-sweetened beverages (≤1, >1–3, >3–5, >5–8, and >8 servings per week), treats (≤2, >2–5, >5–8, >8–14, and >14 servings per week), and toppings (≤2, >2–7, >7–14, >14–28, and >28 servings per week). These categories were informed by restricted cubic spline models in previous publications [29,30]. Intake of specific sugary foods (i.e., pastries, ice cream, sweets, chocolate, table sugar, and honey or jams or marmalades) was also analyzed and categorized into quintiles.

### Assessment of outcome

Cases of incident all-cause dementia and subtypes were identified from the Swedish National Patient Register throughout 2020, including data from both inpatient care and hospital-based outpatient visits registered in the Swedish National Patient Register. Diagnoses were based on the following codes from the ninth and tenth versions of the International Classification of Diseases (ICD): Alzheimer’s disease (ICD-10 and ICD-9 codes F00, G30, 331A/331.0), vascular dementia (F01, 290E/290.4), Parkinson’s disease dementia (F020, G310, 331B/331.1), and unspecified dementia (F03, 290, 294B/294.1, 331C/331.2). Diagnoses until 31 December, 2014, were validated by physicians through a review of medical records, which included evaluations of symptoms, results from cognitive tests and brain imaging, and, when available, cerebrospinal fluid levels of Aβ42 and *p*-τ in accordance with the criteria of the Diagnostic and Statistical Manual of Mental Disorders, Fifth Edition. Diagnoses from 2015 to 2020 were not clinically validated but were obtained from national register codes. These unvalidated cases are still considered 96% reliable for identifying all-cause dementia, though subtype classifications may be less certain [32]. Therefore, analyses of all-cause dementia included both validated (up to 2014) and unvalidated (2015–2020) diagnoses, whereas analyses of dementia subtypes were restricted to validated cases.

### *APOE* genotyping

Individuals were categorized as *APOE* ε4 carriers or noncarrier (ε2 or ε3 alleles). Genotyping was performed using the Illumina NeuroChip array. *APOE* status was available for 26,775 participants (96%), of whom 8092 were ε4 carriers, and 18,683 were noncarriers.

### Statistical analysis

Cox proportional hazards regression models were used to calculate the hazard ratios (HRs) and 95% confidence intervals (CIs) for all-cause dementia and its subtypes in relation to free sugar intake and sugary foods and beverages. Potential confounders were selected based on the 2020 Lancet Commission report on dementia prevention, intervention, and care [7]. Two models were used: model 1 adjusted for age, sex, season of dietary assessment (spring, summer, autumn, or winter), diet method (before and after 1 September, 1994), and energy intake (kcal/d); model 2 additionally adjusted for smoking status (current, former, or never), education (elementary school, primary/secondary school, upper secondary school, further education without a degree, or university degree), leisure-time physical activity (<7.5 MET-h/wk, 7.5–15 MET-h/wk, 15–25 MET-h/wk, 25–50 MET-h/wk, or >50 MET-h/wk), alcohol consumption (g/d), BMI, dietary factors (fruits, vegetables, nuts, processed meat, coffee, saturated fat, and fiber density), Social Network Index (0–5, continuous) [33], prevalent diabetes (yes/no), and antihypertensive medication (yes/no). The dietary factors were selected based on their known associations with sugar intake in this cohort [34] and their potential roles for dementia, particularly for vascular dementia. To assess its influence separately, BMI was introduced as an intermediate step between model 1 and model 2. Linear trend tests were performed by modeling free sugar intake as a continuous variable in the Cox proportional hazards models. Restricted cubic spline analyses were conducted based on model 2 to examine nonlinear associations between free sugar intake and dementia risk. To identify the optimal curve fit, models with 3–7 cutoff points were tested, with the model demonstrating the lowest Akaike information criterion selected. Five knots were used (at percentiles 5, 27.5, 50, 72.5, and 95). Food-specific analyses were conducted to examine associations between the intake of individual sugary foods and beverages and dementia outcomes. For these analyses, participants were categorized into quintiles (Q1–Q5) of intake based on cohort-specific distributions, with the lowest quintile serving as the reference group. Cox proportional hazards models were fitted using model 2 covariate adjustment. In an additional model, all food group variables were mutually adjusted for each other to account for correlated dietary behaviors. *P* for trend was calculated by modeling the intake as a continuous variable.

Effect modification by *APOE* ε4 status (carriers compared with noncarriers) and sex was tested by including multiplicative interaction terms in the Cox models. Several sensitivity analyses were performed to assess the robustness of the findings. First, to reduce the risk of reverse causation, participants who received a dementia diagnosis within 10 y of study start were excluded. Next, participants identified as energy misreporters or those who had made significant dietary changes before the baseline examination were excluded, yielding a sample of 17,369 individuals. Because diabetes is both a potential cause of dietary modification (particularly with respect to sugar intake) and a known risk factor for dementia, participants with prevalent diabetes at baseline were then excluded, leaving a total of 26,552 individuals for analysis. Because vascular dementia shares key etiological pathways with cardiovascular disease and hypertension, we further adjusted for baseline cardiovascular disease and hypertension. All analyses were conducted in R Core Team (version 4.4.1) using the “survival” and “plotRCS” packages.

## Results

### Baseline characteristics

The study population comprised 27,786 individuals aged 45–74 y (mean age of 58 y, 61% females). The mean intake of free sugar was 10.5 E%, with mean weekly consumption of 1.9 servings of sugar-sweetened beverages, 7.1 servings for treats, and 12.5 servings for toppings. Participants with higher free sugar intake were more likely to be females, older, have slightly lower BMI, consume less alcohol, and have lower educational level. In contrast, lower free sugar consumers were likely to misreport energy (primarily underreporting), prior significant dietary change, a history of diabetes, and greater use of antihypertensive medications (Table 1).

**TABLE 1:** Baseline characteristics of the study population by free sugar intake categories

	Free sugar intake categories				
	<7.5 E% (*n =* 6301)	7.5–10 E% (*n =* 6397)	10–12.5 E% (*n =* 6187)	12.5–15 E% (*n =* 4332)	>15 E% (*n =* 4569)
	Mean (SD)				
Age (y)	57.3 (7.3)	58.0 (7.6)	58.4 (7.7)	58.6 (7.8)	58.6 (7.8)
BMI (kg/m^2^)	26.3 (4.2)	25.8 (3.9)	25.6 (3.8)	25.5 (3.8)	25.3 (4.0)
Total energy (kcal/d)	2120 (652)	2267 (642)	2311 (633)	2357 (652)	2375 (658)
Treats (servings/wk)	3.99 (2.99)	6.45 (3.93)	7.72 (4.61)	8.79 (5.36)	9.73 (6.96)
Toppings (servings/wk)	4.46 (4.47)	9.22 (7.24)	12.8 (9.41)	17.1 (12.1)	23.4 (18.8)
SSB (servings/wk)	0.43 (0.99)	0.97 (1.69)	1.59 (2.44)	2.55 (3.29)	5.24 (6.66)
Processed meat (g/d)	44.5 (36.6)	44.4 (34.7)	43.2 (33.6)	42.5 (31.8)	39.4 (30.9)
Coffee (g/d)	560 (437)	519 (387)	509 (374)	488 (352)	504 (401)
Saturated fat (E%)	17.4 (4.4)	17.2 (3.8)	16.9 (3.6)	16.4 (3.6)	15.6 (3.5)
Fiber (g/1000 kcal)	9.72 (3.19)	9.28 (2.62)	9.01 (2.54)	8.78 (2.43)	8.27 (2.58)
Fruits and berries (g/d)	197 (139)	199 (126)	196 (120)	193 (120)	183 (122)
Vegetables (g/d)	204 (119)	187 (97.4)	177 (92.5)	172 (90.6)	158 (87.9)

	Median (IQR)				

Alcohol (g/d)	8.91 (2.11–18.1)	8.17 (2.37–16.2)	7.33 (1.83–15.0)	6.45 (1.29–13.8)	4.57 (0.51–11.9)
Nuts and snacks (g/d)	0 (0–3.33)	0.73 (0–3.83)	0.50 (0–3.60)	0.49 (0–3.57)	0.00 (0–3.41)

	*n* (%)				

Sex					
Male	2644 (42.0)	2578 (40.3)	2334 (37.7)	1654 (38.2)	1726 (37.8)
Female	3657 (58.0)	3819 (59.7)	3853 (62.3)	2678 (61.8)	2843 (62.2)
Season					
Spring	1412 (22.4)	1508 (23.6)	1430 (23.1)	975 (22.5)	969 (21.2)
Summer	2018 (32.0)	1821 (28.5)	1781 (28.8)	1252 (28.9)	1407 (30.8)
Autum	919 (14.6)	900 (14.1)	851 (13.8)	653 (15.1)	717 (15.7)
Winter	1952 (31.0)	2168 (33.9)	2125 (34.3)	1452 (33.5)	1476 (32.3)
Smoking					
Current smoker	1956 (31.0)	1662 (26.0)	1632 (26.4)	1181 (27.3)	1408 (30.8)
Former smoker	2359 (37.4)	2220 (34.7)	2086 (33.7)	1336 (30.8)	1415 (31.0)
Never smoker	1986 (31.5)	2515 (39.3)	2469 (39.9)	1815 (41.9)	1746 (38.2)
Education level					
<9 y	2584 (41.0)	2540 (39.7)	2521 (40.7)	1883 (43.5)	2113 (46.2)
9 y	1586 (25.2)	1673 (26.2)	1746 (28.2)	1115 (25.7)	1158 (25.3)
Upper secondary school	566 (9.0)	589 (9.2)	541 (8.7)	372 (8.6)	407 (8.9)
University (no degree)	555 (8.8)	599 (9.4)	521 (8.4)	379 (8.7)	377 (8.3)
University degree	1010 (16.0)	996 (15.6)	858 (13.9)	583 (13.5)	514 (11.2)
Physical activity (MET-h/wk)					
<7.5	732 (11.6)	554 (8.7)	538 (8.7)	355 (8.2)	511 (11.2)
7.5–15	986 (15.6)	901 (14.1)	908 (14.7)	655 (15.1)	693 (15.2)
15–25	1432 (22.7)	1482 (23.2)	1428 (23.1)	1028 (23.7)	1027 (22.5)
25–50	2240 (35.5)	2439 (38.1)	2339 (37.8)	1535 (35.4)	1535 (33.6)
>50	911 (14.5)	1021 (16.0)	974 (15.7)	759 (17.5)	803 (17.6)
Social Network Index					
0	849 (13.5)	1053 (16.5)	999 (16.1)	657 (15.2)	576 (12.6)
1	1917 (30.4)	2047 (32.0)	1954 (31.6)	1362 (31.4)	1337 (29.3)
2	1869 (29.7)	1883 (29.4)	1808 (29.2)	1295 (29.9)	1436 (31.4)
3	1122 (17.8)	1023 (16.0)	1024 (16.6)	696 (16.1)	774 (16.9)
4	433 (6.9)	324 (5.1)	331 (5.3)	271 (6.3)	348 (7.6)
5	111 (1.8)	67 (1.0)	71 (1.1)	51 (1.2)	98 (2.1)
Prevalent diabetes	648 (10.3)	241 (3.8)	164 (2.7)	90 (2.1)	91 (2.0)
Antihypertensive medication use	1250 (19.8)	1114 (17.4)	1066 (17.2)	751 (17.3)	821 (18.0)
Misreporters of energy intake					
Under report	1508 (23.9)	1007 (15.7)	801 (12.9)	496 (11.4)	473 (10.4)
Accurate	4684 (74.3)	5241 (81.9)	5193 (83.9)	3682 (85.0)	3857 (84.4)
Over report	109 (1.7)	149 (2.3)	193 (3.1)	154 (3.6)	239 (5.2)
Drastic diet changers					
Yes	1952 (31.0)	1494 (23.4)	1339 (21.6)	919 (21.2)	1067 (23.4)
No	4349 (68.9)	4903 (76.6)	4848 (78.4)	3413 (78.8)	3502 (76.6)

Abbreviations: E%, percentage of nonalcoholic energy intake; MET, metabolic equivalent of task; SSB, sugar-sweetened beverages.

Treats: pastries, ice cream, sweets, and chocolate; toppings: table sugar, honey, jams, and marmalades; sugar-sweetened beverages (SSBs): sweetened sodas and fruit drinks, excluding 100% fruit juices; nuts and snacks: nuts and snack foods (chips/crisps and other salty snacks).

### Associations between free sugar intake and dementia risk

**FIGURE 2:**
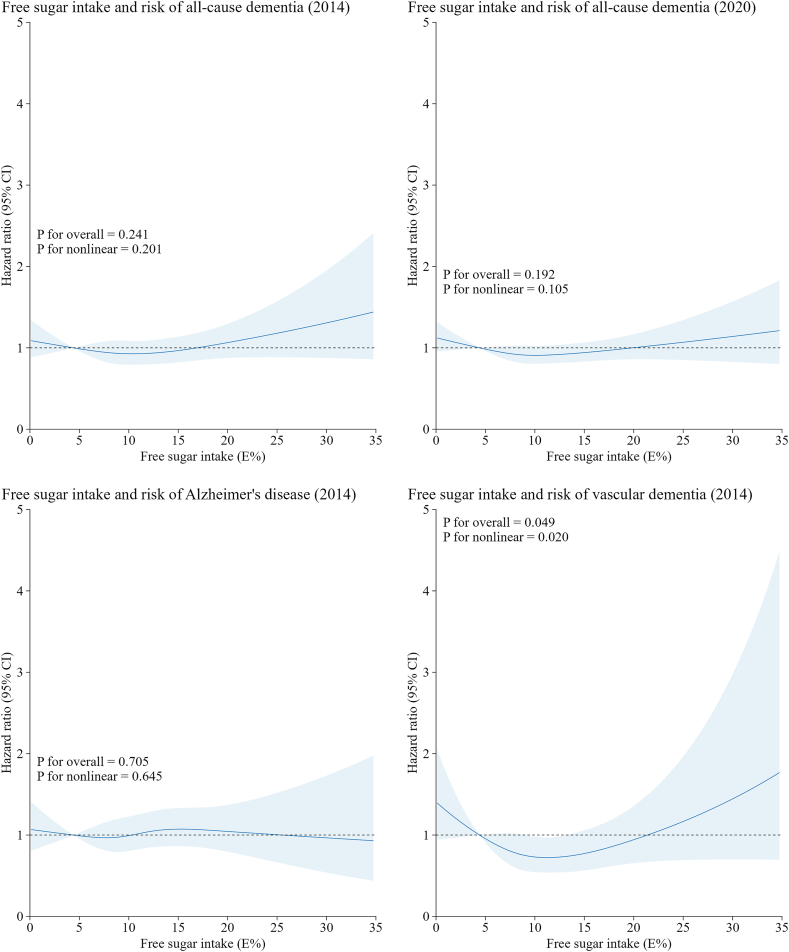
Restricted cubic splines showing dose-response association between free sugar and all-cause dementia, Alzheimer’s disease, and vascular dementia. Adjusted for age, sex, season of dietary assessment, diet method, energy intake, smoking status, educational level, leisure-time physical activity, alcohol consumption (grams per day), BMI, dietary habits (fruits, vegetables, nuts, processed meat, coffee, saturated fat, and fiber density), Social Network Index, prevalent diabetes, and antihypertensive medication use. CI, confidence interval.

Among the 27,786 dementia-free participants, 1928 (6.9%) developed dementia over a median follow-up of 19.7 y, based on validated diagnoses through 2014. Of these cases, 1129 cases were Alzheimer’s disease, and 455 cases were vascular dementia. Including unvalidated register-based diagnoses from 2015 to 2020, the total number of dementia cases increased to 3224 (11.6%) over a median follow-up of 24.9 y. In fully adjusted models, no significant linear or nonlinear association was observed between free sugar intake and risk of all-cause dementia (Figure 2 and Table 2). Similarly, there was no association between free sugar intake and Alzheimer’s disease risk (Table 3). However, for vascular dementia, a U-shaped association was identified. Participants in the 10–12.5 E% intake category had a significantly lower risk (HR: 0.69; 95% CI: 0.51, 0.93) compared with the reference category (<7.5 E%) (Table 3). This nonlinear association was statistically significant (*P* = 0.02; Figure 2).

**TABLE 2:** Associations between intake of free sugar and sugar sources and risk of all-cause dementia

	All-cause dementia (2014)			All-cause dementia (2020)		
	Cases/person-years	Model 1	Model 2	Cases/person-years	Model 1	Model 2
Free sugar intake						
<7.5 E%	379/114,126	Ref	Ref	661/135,884	Ref	Ref
7.5–10 E%	455/118,325	1.00 (0.87–1.14)	1.03 (0.90–1.19)	756/141,664	0.94 (0.85–1.05)	0.97 (0.87–1.08)
10–12.5 E%	395/113,689	0.84 (0.72–0.96)	0.87 (0.75–1.01)	696/135,589	0.86 (0.77–0.96)	0.89 (0.79–0.99)
12.5–15 E%	350/78,655	1.05 (0.91–1.22)	1.08 (0.93–1.27)	536/93,514	0.95 (0.85–1.07)	0.98 (0.86–1.10)
>15 E%	349/81,939	1.04 (0.89–1.20)	1.06 (0.89–1.25)	575/96,931	1.02 (0.91–1.15)	1.03 (0.91–1.17)
*P*-trend	—	0.40	0.34	—	0.67	0.64
Treats						
≤2 servings/wk	177/58,606	Ref	Ref	316/69,306	Ref	Ref
2–5 servings/wk	500/141,313	1.02 (0.86–1.21)	1.04 (0.87–1.24)	846/169,253	0.96 (0.85–1.10)	0.98 (0.86–1.12)
5–8 servings/wk	523/132,526	1.00 (0.85–1.19)	1.04 (0.87–1.24)	868/158,379	0.94 (0.82–1.07)	0.97 (0.85–1.11)
8–14 servings/wk	531/129,577	0.93 (0.78–1.11)	0.96 (0.80–1.15)	868/153,891	0.87 (0.76–0.99)	0.90 (0.78–1.03)
>14 servings/wk	197/44,713	1.06 (0.85–1.32)	1.08 (0.86–1.36)	326/52,753	1.00 (0.85–1.18)	1.02 (0.86–1.22)
*P*-trend	—	0.71	0.79	—	0.34	0.50
Toppings						
≤2 servings/wk	220/71,336	Ref	Ref	373/85,547	Ref	Ref
2–7 servings/wk	491/138,447	0.96 (0.82–1.13)	0.98 (0.84–1.15)	829/166,077	0.97 (0.86–1.10)	0.99 (0.87–1.12)
7–14 servings/wk	524/134,546	0.89 (0.76–1.04)	0.91 (0.77–1.07)	897/160,526	0.93 (0.82–1.05)	0.96 (0.84–1.08)
14–28 servings/wk	485/113,406	0.92 (0.78–1.08)	0.93 (0.78–1.11)	790/134,021	0.93 (0.81–1.05)	0.95 (0.83–1.09)
>28 servings/wk	208/48,999	1.08 (0.88–1.33)	1.06 (0.85–1.32)	335/57,411	1.06 (0.91–1.25)	1.06 (0.90–1.26)
*P*-trend	—	0.09	0.17	—	0.14	0.19
Sugar-sweetened beverages						
≤1 servings/wk	1204/298,639	Ref	Ref	1955/355,167	Ref	Ref
1–3 servings/wk	334/104,888	0.88 (0.78–0.99)	0.87 (0.77–0.99)	607/125,858	0.95 (0.86–1.04)	0.94 (0.86–1.03)
3–5 servings/wk	164/43,807	1.00 (0.85–1.17)	0.98 (0.83–1.15)	275/52,265	1.01 (0.89–1.15)	1.00 (0.88–1.13)
5–8 servings/wk	116/30,180	1.04 (0.86–1.26)	1.02 (0.84–1.24)	203/35,877	1.13 (0.98–1.31)	1.11 (0.96–1.29)
>8 servings/wk	110/29,220	1.05 (0.86–1.28)	1.00 (0.81–1.22)	184/34,414	1.10 (0.94–1.28)	1.06 (0.90–1.24)
*P*-trend	—	0.44	0.78	—	0.20	0.19

Abbreviations: E%, percentage of nonalcoholic energy intake; Ref, reference.

Cox proportional hazard models were used to investigate associations between free sugar intake and all-cause dementia (yes or no) by 2014 (validated register data), and 2020 (validated register diagnoses until 2014, and unvalidated diagnoses between 2015–2020).

Model 1 was adjusted for age, sex, season of dietary assessment, diet method, and energy intake.

Model 2 was adjusted for age, sex, season of dietary assessment, diet method, energy intake, smoking status, educational level, leisure-time physical activity, alcohol consumption (grams per day), BMI, dietary habits (fruits, vegetables, nuts, processed meat, coffee, saturated fat, and fiber density), Social Network Index, prevalent diabetes, and antihypertensive medication use.

Sugar-sweetened beverages (SSBs), sweetened sodas and fruit drinks, excluding 100% fruit juices; Treats, pastries, ice cream, sweets, and chocolate; Toppings, table sugar, honey, jams, and marmalades.

**TABLE 3:** Associations between intake of free sugar and sugar sources and risk of Alzheimer’s disease and vascular dementia

	Alzheimer’s disease			Vascular dementia		
	Cases/person-years	Model 1	Model 2	Cases/person-years	Model 1	Model 2
Free sugar intake						
<7.5 E%	208/114,574	Ref	Ref	111/115,042	Ref	Ref
7.5–10 E%	263/118,932	1.04 (0.87–1.25)	1.07 (0.89–1.29)	96/119,717	0.71 (0.54–0.93)	0.79 (0.60–1.05)
10–12.5 E%	249/114,103	0.95 (0.79–1.15)	0.98 (0.81–1.20)	84/114,790	0.61 (0.46–0.81)	0.69 (0.51–0.93)
12.5–15 E%	217/79,090	1.17 (0.96–1.42)	1.22 (0.99–1.50)	80/79,677	0.82 (0.61–1.09)	0.90 (0.66–1.23)
>15 E%	192/82,398	1.02 (0.83–1.24)	1.08 (0.87–1.35)	84/82,852	0.85 (0.64–1.14)	0.88 (0.64–1.22)
*P*-trend	—	0.85	0.47	—	0.97	0.86
Treats						
≤2 servings/wk	93/58,838	Ref	Ref	54/59,051	Ref	Ref
2–5 servings/wk	301/141,879	1.14 (0.90–1.44)	1.15 (0.91–1.46)	118/142,619	0.80 (0.58–1.10)	0.86 (0.62–1.19)
5–8 servings/wk	318/133,196	1.13 (0.90–1.43)	1.15 (0.90–1.46)	121/133,986	0.77 (0.55–1.06)	0.86 (0.62–1.21)
8–14 servings/wk	307/130,201	1.01 (0.79–1.28)	1.02 (0.79–1.30)	120/131,131	0.69 (0.49–0.96)	0.80 (0.56–1.13)
>14 servings/wk	110/44,982	1.12 (0.83–1.50)	1.14 (0.84–1.55)	42/45,291	0.73 (0.47–1.12)	0.80 (0.51–1.27)
*P*-trend	—	0.44	0.53	—	0.25	0.52
Toppings						
≤2 servings/wk	122/71,610	Ref	Ref	62/71872	Ref	Ref
2–7 servings/wk	296/139,026	1.03 (0.83–1.27)	1.04 (0.84–1.29)	111/139,914	0.78 (0.57–1.07)	0.84 (0.61–1.15)
7–14 servings/wk	322/135,123	0.99 (0.80–1.22)	1.00 (0.81–1.25)	116/136,021	0.69 (0.50–0.94)	0.76 (0.55–1.05)
14–28 servings/wk	283/114,017	1.01 (0.81–1.26)	1.03 (0.82–1.30)	104/114,718	0.65 (0.47–0.90)	0.72 (0.51–1.01)
>28 servings/wk	106/49,320	1.09 (0.82–1.44)	1.13 (0.84–1.52)	62/49,553	1.02 (0.69–1.50)	1.02 (0.68–1.54)
*P*-trend	—	0.27	0.16	—	0.29	0.43
Sugar-sweetened beverages						
≤1 servings/wk	700/300,142	Ref	Ref	291/301,934	Ref	Ref
1–3 servings/wk	205/105,268	0.94 (0.80–1.09)	0.93 (0.80–1.09)	73/105,822	0.81 (0.63–1.05)	0.82 (0.63–1.06)
3–5 servings/wk	96/44,028	1.02 (0.83–1.27)	1.02 (0.82–1.27)	34/44,331	0.84 (0.59–1.20)	0.83 (0.58–1.20)
5–8 servings/wk	70/30,295	1.11 (0.87–1.43)	1.12 (0.87–1.44)	26/30,500	0.96 (0.64–1.44)	0.92 (0.61–1.39)
>8 servings/wk	58/29,362	0.99 (0.75–1.30)	1.01 (0.76–1.33)	31/29,490	1.19 (0.82–1.74)	1.06 (0.72–1.57)
*P*-trend	—	0.60	0.52	—	0.60	0.91

Abbreviations: E%, percentage of nonalcoholic energy intake; Ref, reference.

Cox proportional hazard models were used to investigate associations between free sugar intake and all-cause dementia (yes or no) by 2014 (validated register data), and 2020 (validated register diagnoses until 2014, and unvalidated diagnoses between 2015–2020).

Model 1 was adjusted for age, sex, season of dietary assessment, diet method, and energy intake.

Model 2 was adjusted for age, sex, season of dietary assessment, diet method, energy intake, smoking status, educational level, leisure-time physical activity, alcohol consumption (g/d), BMI, dietary habits (fruits, vegetables, nuts, processed meat, coffee, saturated fat, and fiber density), Social Network Index, prevalent diabetes, and antihypertensive medication use.

Sugar-sweetened beverages (SSBs): sweetened sodas and fruit drinks, excluding 100% fruit juices; Treats, pastries, ice cream, sweets, and chocolate; toppings, table sugar, honey, jams, and marmalades.

### Associations between sugary food and beverage intake and dementia risk

**FIGURE 3:**
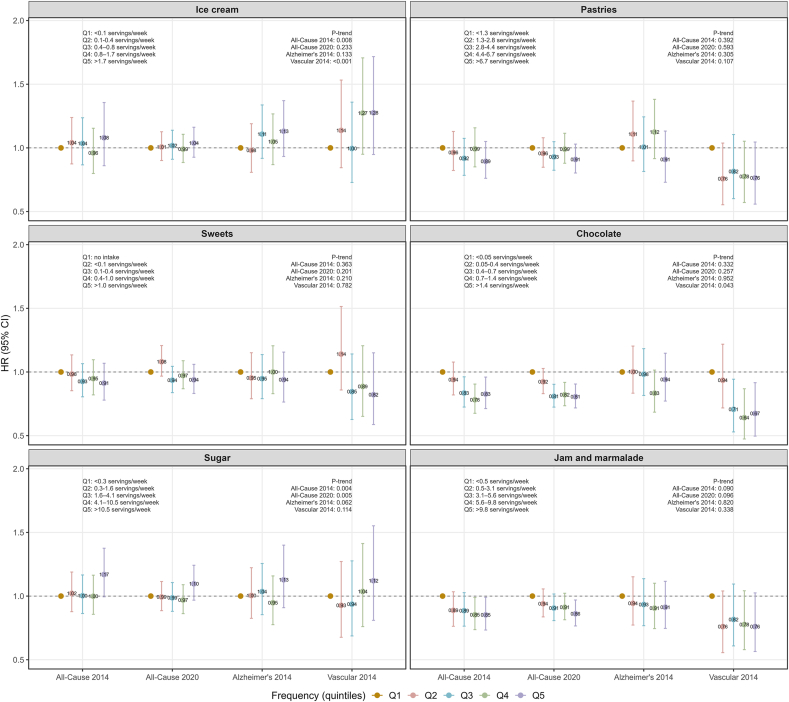
Associations between specific sugar sources and risk of all-cause dementia, Alzheimer’s disease and vascular dementia. Adjusted for age, sex, season of dietary assessment, diet method, energy intake, smoking status, educational level, leisure-time physical activity, alcohol consumption (grams per day), BMI, dietary habits (fruits, vegetables, nuts, processed meat, coffee, saturated fat, and fiber density), Social Network Index, prevalent diabetes, and antihypertensive medication use. *P*-trend was calculated by modeling the intake as a continuous variable. CI, confidence interval; HR, hazard ratio.

No significant associations were found between the consumption of treats, toppings, or sugar-sweetened beverages and risk of all-cause dementia, Alzheimer’s disease, or vascular dementia (TABLE 2, TABLE 3). Figure 3 illustrates the associations for specific sugary foods (ice cream, pastries, sweets, chocolate, sugar, and jam/marmalade). A linear positive association was observed between ice cream intake and all-cause dementia diagnosed up to 2014 (*P* = 0.008), as well as vascular dementia (*P* < 0.001). A linear positive association was also found between table sugar intake and all-cause dementia (*P* = 0.004). In contrast, higher chocolate intake was associated with a lower risk of all-cause dementia and vascular dementia, with statistically significant associations for Q3, Q4, and Q5 compared with Q1. The intake level in Q3 corresponded to 0.4 servings per week. For example, the HR for Q5 (>1.4 servings per week) compared with Q1 (<0.005 servings per week) was 0.81 (95% CI: 0.72, 0.91) for all-cause dementia. For vascular dementia, the HR was 0.67 (95% CI: 0.50, 0.92) for Q5 compared with Q1. Higher intake of jam/marmalade was also associated with a lower risk of all-cause dementia [HR Q5 (>9.8 servings per week) compared with Q1 (<0.5 servings per week): 0.86, 95% CI: 0.77, 0.97]. No significant associations were found between these sugary foods and Alzheimer’s disease risk (Figure 3 and Supplementary Tables 1 and 2).

### Interaction and sensitivity analysis

There were no significant linear or nonlinear associations between free sugar intake and dementia risk when stratified by sex (Supplementary Table 3). No statistically significant interactions were observed between free sugar intake and APOE *ε4* status for any dementia outcome (Supplementary Figure 1 and Supplementary Table 4). However, stratified analyses revealed a significant nonlinear U-shaped association (*P*-nonlinear = 0.039) between free sugar intake and all-cause dementia among noncarriers of *APOE ε4* up to 2020, but not among carriers, suggesting a potential genetic effect modification (Supplementary Figure 1).

Results were materially unchanged after excluding dementia cases diagnosed within 10 y after baseline (Supplementary Table 5), indicating that the main findings are robust and unlikely to be explained by reverse causation. Sensitivity analyses excluding energy misreporters and participants with dietary changes before baseline yielded results consistent with the main findings, although some variations in effect size and significance were noted (Supplementary Table 6). Excluding individuals with diabetes at baseline did not materially change the association (Supplementary Table 7). Because vascular dementia shares key etiological pathways with cardiovascular disease and hypertension, we further examined whether the observed U-shaped association for free sugar could be explained by pre-existing cardiovascular comorbidity. After additional adjustment for baseline cardiovascular disease and hypertension, the U-shaped association between free sugar intake and vascular dementia remained evident (*P*-nonlinear = 0.016).

## Discussion

This study examined the association between free sugar intake and the risk of all-cause dementia, Alzheimer’s disease, and vascular dementia in a large population-based cohort. Although no significant associations were observed between free sugar intake and the risk of all-cause dementia or Alzheimer’s disease, a U-shaped relationship was identified for vascular dementia, with moderate free sugar intake (10–12.5 E%) associated with the lowest risk. Furthermore, sugar-sweetened beverage intake was not associated with dementia risk, whereas higher consumption of chocolate and jam/marmalade was linked to lower risk of all-cause dementia and vascular dementia. No significant interactions were found between free sugar intake and *APOE* ε4 status; however, stratified analyses suggested a nonlinear association among noncarriers, which was not evident in carriers.

Few studies have examined the association between free or added sugar intake and dementia risk. Schaefer et al. [16] identified a J-shaped relationship between free sugar intake and dementia risk, indicating that moderate intake levels (around 9% of total energy) were associated with lower risks than either low or high intakes. The U-shaped association for vascular dementia observed in the present study aligns with findings from Swedish cohorts on cardiovascular outcomes, where both low and high-sugar intake were linked to increased risk [30,34]. The mechanisms may involve glucose availability for normal brain function at moderate intake and vascular damage at high intake. Consequently, the same metabolic disturbances that elevate cardiovascular disease risk at higher sugar intakes—insulin resistance, chronic inflammation, and endothelial dysfunction—might also increase the risk of vascular dementia [2]. However, the association persisted after additional adjustment for baseline cardiovascular disease and hypertension, with moderate free sugar intake remaining associated with a lower risk of vascular dementia. These findings suggest that the observed relationship is not solely driven by overt cardiovascular disease or hypertension, although residual confounding and limited case numbers in subgroup analyses cannot be excluded. An additional explanation for the elevated risk observed at low intakes may be related to dietary misreporting. Underreporting is common in dietary assessments, particularly among individuals with overweight, diabetes, or health-conscious behaviors, which may introduce bias into the lowest intake categories. Although sensitivity analyses excluding misreporters and individuals with major dietary changes before the baseline were performed, residual effects may persist. Thus, the elevated dementia risk at low sugar intakes should be interpreted with caution.

The null association for sugar-sweetened beverages in the present study partially diverges from previous reports. For example, results from the Framingham Heart Study (*n* = 3702) found that higher consumption of sugar-sweetened beverages (1–7 servings per week compared with no intake) was associated with an increased risk of both all-cause dementia and Alzheimer’s disease [35]. In the UK Biobank, Chen et al. [36] observed a 34% higher dementia risk among participants consuming >2 servings of sugar-sweetened beverages per day (>500 mL/d), compared with nonconsumers. In addition, Schaefer et al. [16] reported a linear association between free sugars from beverages and dementia risk, particularly at intake levels around 10 E%—whereas no such relationship was found for free sugars from solid foods. Several limitations in existing literature were highlighted in the meta-analysis by Sun et al. [37], including low intake levels in certain populations (notably in Asia), inconsistent definitions of sugar-sweetened beverages, and heterogeneous study designs. In this Swedish population, only ∼6% of participants consumed >8 servings of sugar-sweetened beverages per week, potentially limiting statistical power - particularly if higher intake thresholds are required to activate metabolic or vascular mechanisms relevant to dementia. By contrast, larger cohorts in the United Kingdom and the United States include more individuals who consume ≥2 servings per day [35,36]. Thus, the null finding observed in the present study may reflect both lower absolute intake and distinct beverage consumption patterns within the Swedish dietary context. Future research involving populations with higher and more varied sugar-sweetened beverage consumption could help clarify whether the absence of association observed is specific to a low intake setting. Differences in cohort demographics, lifestyle factors, and dietary assessment methods may also contribute to variability across studies.

Additional support for the source-dependent effect of sugar comes from a recent systematic review and meta-analysis of 65 intervention studies, 9 cross-sectional studies, and 3 prospective cohort studies with sample sizes ranging from 11 to 6387 participants [38]. Across all cohort studies and 8 of the 9 cross-sectional studies in the review, significant positive associations emerged between added sugar intake and cognitive impairment risk, whereas 4 studies noted reduced risk tied to fructose-containing foods such as whole fruits. These findings align with research indicating that short-term sugar consumption can temporarily enhance performance (“glucose facilitation”) under certain conditions, yet excessive or long-term intake—particularly from sources high in refined sugars or high-fructose corn syrup—can be detrimental to vascular and metabolic health. Conversely, fruit-based sugars may exert protective effects, possibly owing to accompanying fiber or other nutrients rather than fructose alone. The protective association for jam and marmalade aligns with findings from the same cohort, indicating a lower risk of type 2 diabetes [39], possibly because jam and marmalade are often consumed with healthy foods such as oatmeal or fiber-rich bread. Although observational, our finding of a lower risk of dementia with higher chocolate intake aligns with prior evidence linking chocolate consumption to reduced cognitive decline and lower dementia mortality [17,40,41]. These associations have been hypothesized to involve cocoa flavonoids and other bioactive compounds that influence vascular and neurocognitive pathways [42]. Overall, evidence continues to point toward the importance of both sugar source and quantity in determining potential risks and benefits for cardiometabolic and cognitive health.

Although vascular dementia is the second most common form of dementia globally, it remains underexplored in relation to dietary risk factors. A recent systematic review by Griffiths et al. [43] synthesized evidence from 16 prospective cohort studies and found that higher intake of fruits and vegetables, tea and coffee, and moderate alcohol consumption were associated with lower vascular dementia risk, whereas high intake of ultraprocessed foods, including sugar-sweetened beverages, and processed meats, was associated with increased risk. However, the authors emphasize that the number of studies on specific dietary exposures remains limited, highlighting a need for further research to establish more targeted dietary recommendations for vascular dementia prevention.

Although no significant interaction emerged between free sugar intake and *APOE* ε4 status, stratified analyses suggested a nonlinear association only in noncarriers. This aligns with prior studies indicating that *APOE* ε4 carriers often respond less favorably to dietary interventions, possibly due to altered lipid and glucose metabolism [44]. For instance, Seaks et al. [45] demonstrated that the cognitive impact of fat intake varied by *APOE* genotype, with *APOE* ε4 carriers showing less pronounced benefits from dietary modifications. Although the formal interaction test was not significant, this discrepancy may reflect known differences in lipid and glucose metabolism attributable to the *APOE* ε4 allele. Previous work suggests that *APOE* ε4 carriers often respond less favorably to dietary modifications, potentially due to altered neuronal glucose uptake [45]. The smaller sample size among *APOE* ε4 carriers may have limited power to detect effect modification. Future research with larger cohorts would help clarify whether *APOE* ε4 truly modifies the relationship between sugar intake and dementia risk. Nonetheless, the observed nonlinear association among noncarriers aligns with the notion that genetic background may influence dietary sensitivity.

Major strengths of this study include the large, well-characterized cohort, long follow-up, and use of validated dementia diagnoses by trained physicians. The inclusion of unvalidated diagnoses (2015–2020) increased case numbers, and previous work shows that all-cause dementia diagnoses have a 96% validity rate. However, for Alzheimer’s disease and vascular dementia, we only used the validated data because of reclassification rates of ∼40% of specific dementia diagnoses during validation [32]. Robust sensitivity analyses excluding potential energy misreporters, those with dietary changes, and prevalent diabetes cases, add confidence to the results. Nevertheless, some limitations must be acknowledged. First, self-reported dietary data are prone to recall bias and misreporting. Still, the MDC dietary method captured both habitual and recent intakes, and previous validation studies have shown good reliability and reproducibility [28,46]. The dietary data were only collected at baseline; however, previous research has demonstrated rather stable dietary habits in participants of similar age to those in our study [47,48]. Although we adjusted for a wide range of confounders and conducted several sensitivity analyses, residual confounding cannot be ruled out. Finally, generalizability may be limited to Nordic populations with distinct dietary habits and patterns, and replication in other settings is necessary.

In this study, we observed that moderate sugar intake may be associated with the lowest risk of vascular dementia, whereas no associations were observed for all-cause dementia or Alzheimer’s disease. We also observed higher chocolate and jam/marmalade intake were associated with lower dementia risk; however, these findings are based on observational data and should be interpreted cautiously. These results challenge the common assumption that all sugar is uniformly detrimental and highlight the importance of considering sugar dose and source in dietary guidelines and public health strategies. Given the observational nature of this study, causality cannot be established. Nonetheless, these findings provide foundation for future research exploring subtype-specific effects, gene-diet interactions, and mechanisms. Such insights could inform dietary recommendations for cognitive health and dementia prevention.

## Author contributions

The authors’ responsibilities were as follows – JA, ES: conceived and designed the study; NZ: performed the statistical analyses; NZ, JA: wrote the manuscript; KN, OH: provided essential materials; and all authors: contributed to the interpretation of the results and revision of the manuscript, and read and approved the final manuscript.

## Data availability

The data that support the findings of this study are available from “The Malmö Cohorts” at Lund University with the permission of the MDC Steering Committee (https://www.malmo-kohorter.lu.se/malmo-cohorts).

## Declaration of generative AI and AI-assisted technologies in the manuscript preparation process

During the preparation of this work, the authors used Chat GPT to improve readability and language of the work. After using this tool, the authors reviewed and edited the content as needed and take full responsibility for the content of the published article.

## Funding

Work at the Clinical Memory Research Unit was supported by the European Research Council (ADG-101096455), Alzheimer’s Association (ZEN24-1069572, SG-23-1061717), GHR Foundation, Swedish Research Council (2022-00775), ERA PerMed (ERAPERMED2021-184), Knut and Alice Wallenberg foundation (2022-0231), Strategic Research Area MultiPark (Multidisciplinary Research in Parkinson’s disease) at Lund University, Swedish Alzheimer Foundation (AF-980907), Swedish Brain Foundation (FO2021-0293, #FO2024-0284), Parkinson foundation of Sweden (1412/22), Cure Alzheimer’s fund, Rönström Family Foundation, Konung Gustaf V:s och Drottning Victorias Frimurarestiftelse, Skåne University Hospital Foundation (2020-O000028), Regionalt Forskningsstöd (2022-1259) and Swedish federal government under the ALF agreement (2022-Projekt0080).

Work in the Nutritional Epidemiology research group was supported by the Swedish Research Council (2020-01412), Heart and Lung foundation (20220444 and 2022662), Påhlsson foundation, and Swedish federal government under the ALF agreement (2022-Projekt0171).

## Conflict of interest

SP has acquired research support (for the institution) from Avid and ki elements through Alzheimer’s Drug Discovery Foundation (ADDF). In the past 2 y, he has received consultancy or speaker fees from Bioartic, Eisai, Eli Lilly, Novo Nordisk, and Roche. OH is an employee of Lund University and Eli Lilly. All other authors report no conflicts of interest.
