# Supplementary material for: Free Sugar Intake and Dementia Risk: A Swedish Cohort Study on Dietary Sources and Dementia Subtypes
Source: J Nutr. 2026 Apr 1;156(6):101518. doi: 10.1016/j.tjnut.2026.101518 (PMC13279334; doi:10.1016/j.tjnut.2026.101518)
Supplement: Multimedia component 1 [file mmc1.docx]

**Supplemental materials**

Zhang et al: Free sugar intake and dementia risk: a Swedish cohort study on dietary sources and dementia subtypes

**Supplementary Table 1**. Associations between intake of specific sugar sources and risk of all-cause dementia.

|  |  | All-cause dementia 2020 | | | | All-cause dementia 2014 | | | |
| --- | --- | --- | --- | --- | --- | --- | --- | --- | --- |
| Exposure | Category | Cases/py | Model 1 | Model 2 | Model 3 | Cases/py | Model 1 | Model 2 | Model 3 |
| Icecream | <0.1 servings/week | 615/115453 | Ref | Ref | Ref | 363/97967 | Ref | Ref | Ref |
|  | 0.1-0.4 servings/week | 636/120865 | 0.99 (0.89-1.11) | 1.01 (0.90-1.13) | 1.01 (0.91-1.13) | 365/101372 | 1.00 (0.86-1.15) | 1.01 (0.87-1.17) | 1.02 (0.88-1.18) |
|  | 0.4–0.8 servings/week | 641/122890 | 1.00 (0.89-1.11) | 1.02 (0.91-1.14) | 1.03 (0.92-1.15) | 378/102788 | 1.04 (0.90-1.20) | 1.06 (0.91-1.22) | 1.07 (0.93-1.24) |
|  | 0.8–1.7 servings/week | 664/122143 | 0.96 (0.86-1.07) | 0.99 (0.89-1.11) | 1.00 (0.89-1.12) | 411/102027 | 1.05 (0.91-1.21) | 1.08 (0.94-1.25) | 1.10 (0.95-1.26) |
|  | >1.7 servings/week | 668/122230 | 1.00 (0.90-1.12) | 1.04 (0.93-1.16) | 1.05 (0.94-1.18) | 411/102581 | 1.09 (0.94-1.26) | 1.13 (0.98-1.31) | 1.15 (1.00-1.33) |
|  | *P* _trend_ |  | 0.455 | 0.233 | 0.166 |  | 0.024 | 0.008 | 0.005 |
| Pastries | <1.3 servings/week | 507/118475 | Ref | Ref | Ref | 294/99295 | Ref | Ref | Ref |
|  | 1.3-2.8 servings/week | 576/122796 | 0.95 (0.84-1.07) | 0.96 (0.85-1.08) | 0.96 (0.86-1.09) | 335/102123 | 0.96 (0.82-1.12) | 0.96 (0.82-1.13) | 0.97 (0.83-1.14) |
|  | 2.8-4.4 servings/week | 625/123200 | 0.92 (0.81-1.03) | 0.93 (0.82-1.05) | 0.94 (0.84-1.06) | 366/102823 | 0.91 (0.78-1.06) | 0.92 (0.78-1.07) | 0.93 (0.80-1.09) |
|  | 4.4-6.7 servings/week | 754/120852 | 0.97 (0.87-1.09) | 0.99 (0.88-1.11) | 1.00 (0.89-1.13) | 462/101964 | 0.99 (0.85-1.14) | 0.99 (0.85-1.16) | 1.01 (0.87-1.18) |
|  | >6.7 servings/week | 762/118259 | 0.90 (0.79-1.01) | 0.91 (0.80-1.03) | 0.93 (0.82-1.05) | 471/100530 | 0.89 (0.77-1.04) | 0.89 (0.76-1.05) | 0.91 (0.78-1.07) |
|  | *P* _trend_ |  | 0.459 | 0.593 | 0.810 | P_trend |  | 0.397 | 0.392 |
| Sweets | no intake | 680/118748 | Ref | Ref | Ref | 426/100401 | Ref | Ref | Ref |
|  | <0.1 servings/week | 767/115792 | 1.12 (1.01-1.24) | 1.08 (0.97-1.21) | 1.08 (0.97-1.21) | 448/99052 | 1.01 (0.88-1.15) | 0.98 (0.85-1.13) | 0.98 (0.85-1.13) |
|  | 0.1-0.4 servings/week | 656/122371 | 0.96 (0.86-1.07) | 0.94 (0.84-1.04) | 0.94 (0.84-1.04) | 403/102562 | 0.95 (0.83-1.09) | 0.93 (0.80-1.07) | 0.93 (0.80-1.07) |
|  | 0.4-1.0 servings/week | 612/122872 | 1.00 (0.90-1.12) | 0.97 (0.87-1.09) | 0.98 (0.87-1.09) | 364/102439 | 0.98 (0.85-1.12) | 0.95 (0.82-1.10) | 0.95 (0.82-1.10) |
|  | >1.0 servings/week | 509/123799 | 0.99 (0.88-1.11) | 0.94 (0.83-1.06) | 0.94 (0.84-1.07) | 287/102280 | 0.95 (0.82-1.11) | 0.91 (0.78-1.07) | 0.91 (0.78-1.07) |
|  | *P* _trend_ |  | 0.420 | 0.201 | 0.237 |  | 0.604 | 0.363 | 0.387 |
| Chocolate | <0.05 servings/week | 680/114153 | Ref | Ref | Ref | 421/97238 | Ref | Ref | Ref |
|  | 0.05-0.4 servings/week | 689/120342 | 0.90 (0.81-1.00) | 0.92 (0.83-1.03) | 0.92 (0.83-1.03) | 419/100916 | 0.92 (0.80-1.05) | 0.94 (0.82-1.08) | 0.94 (0.82-1.08) |
|  | 0.4–0.7 servings/week | 618/123188 | 0.79 (0.70-0.88) | 0.81 (0.72-0.90) | 0.81 (0.72-0.90) | 374/102802 | 0.81 (0.70-0.93) | 0.83 (0.72-0.96) | 0.84 (0.73-0.97) |
|  | 0.7–1.4 servings/week | 626/123660 | 0.79 (0.71-0.89) | 0.82 (0.73-0.92) | 0.82 (0.74-0.92) | 346/103182 | 0.75 (0.65-0.87) | 0.78 (0.68-0.91) | 0.78 (0.68-0.91) |
|  | >1.4 servings/week | 611/122238 | 0.78 (0.70-0.88) | 0.81 (0.72-0.91) | 0.81 (0.72-0.91) | 368/102596 | 0.80 (0.69-0.92) | 0.83 (0.71-0.96) | 0.83 (0.71-0.97) |
|  | *P* _trend_ |  | 0.143 | 0.257 | 0.331 |  | 0.202 | 0.332 | 0.378 |
| Sugar | <0.3 servings/week | 552/122554 | Ref | Ref | Ref | 310/102122 | Ref | Ref | Ref |
|  | 0.3-1.6 servings/week | 634/123442 | 0.98 (0.87-1.10) | 0.99 (0.89-1.11) | 1.00 (0.89-1.12) | 373/103005 | 1.01 (0.87-1.17) | 1.02 (0.88-1.19) | 1.03 (0.88-1.19) |
|  | 1.6–4.1 servings/week | 694/122050 | 0.97 (0.87-1.09) | 0.99 (0.88-1.11) | 0.99 (0.88-1.11) | 412/102199 | 0.99 (0.85-1.14) | 1.00 (0.86-1.17) | 1.01 (0.87-1.17) |
|  | 4.1–10.5 servings/week | 694/119628 | 0.95 (0.84-1.06) | 0.97 (0.86-1.09) | 0.97 (0.86-1.09) | 424/100865 | 0.98 (0.84-1.14) | 1.00 (0.86-1.16) | 1.01 (0.86-1.17) |
|  | >10.5 servings/week | 650/115908 | 1.09 (0.96-1.23) | 1.10 (0.97-1.24) | 1.09 (0.96-1.24) | 409/98544 | 1.17 (1.00-1.36) | 1.17 (0.99-1.38) | 1.17 (0.99-1.38) |
|  | *P* _trend_ |  | 0.001 | 0.005 | 0.009 |  | 0.001 | 0.004 | 0.005 |
| Jam and marmalade | <0.5 servings/week | 560/118666 | Ref | Ref | Ref | 343/99743 | Ref | Ref | Ref |
|  | 0.5-3.1 servings/week | 598/124085 | 0.92 (0.82-1.03) | 0.94 (0.84-1.06) | 0.94 (0.84-1.06) | 339/103035 | 0.87 (0.74-1.01) | 0.89 (0.76-1.03) | 0.89 (0.77-1.04) |
|  | 3.1–5.6 servings/week | 640/120458 | 0.89 (0.79-0.99) | 0.91 (0.81-1.02) | 0.91 (0.81-1.02) | 384/101126 | 0.87 (0.75-1.01) | 0.89 (0.76-1.03) | 0.89 (0.77-1.04) |
|  | 5.6–9.8 servings/week | 707/120364 | 0.89 (0.80-1.00) | 0.91 (0.81-1.02) | 0.92 (0.82-1.03) | 414/101207 | 0.84 (0.73-0.97) | 0.85 (0.74-0.99) | 0.86 (0.74-1.00) |
|  | >9.8 servings/week | 719/120007 | 0.84 (0.75-0.94) | 0.86 (0.77-0.97) | 0.87 (0.77-0.98) | 448/101624 | 0.84 (0.73-0.97) | 0.85 (0.73-0.99) | 0.87 (0.75-1.01) |
|  | *P* _trend_ |  | 0.045 | 0.096 | 0.140 |  | 0.066 | 0.090 | 0.154 |

Cox proportional hazard models were used to investigate associations between free sugar intake and all-cause dementia (yes/no) by 2014 (validated register data), and 2020 (validated register diagnoses until 2014, and unvalidated diagnoses between 2015-2020). Model 1 was adjusted for age, sex, season of dietary assessment, diet method, and energy intake. Model 2 was adjusted for age, sex, season of dietary assessment, diet method, energy intake, smoking status, educational level, leisure-time physical activity, alcohol consumption (g/day), body mass index, dietary habits (fruits, vegetables, nuts, processed meat, coffee, saturated fat, and fiber density), Social Network Index, prevalent diabetes, and anti-hypertensive medication use. Model 3 was adjusted for age, sex, season of dietary assessment, diet method, energy intake, smoking status, educational level, leisure-time physical activity, alcohol consumption (g/day), body mass index, dietary habits (fruits, vegetables, nuts, processed meat, coffee, saturated fat, and fiber density), Social Network Index, prevalent diabetes, anti-hypertensive medication use, and other sugar sources.

**Supplementary Table 2**. Associations between intake of specific sugar sources and risk of Alzheimer’s disease and vascular dementia.

|  |  | **Alzheimer’s disease** | | | | **Vascular dementia** | | | |
| --- | --- | --- | --- | --- | --- | --- | --- | --- | --- |
| Exposure | Category | Cases/py | Model 1 | Model 2 | Model 3 | Cases/py | Model 1 | Model 2 | Model3 |
| Icecream | <0.1 servings/week | 209/98407 | Ref | Ref | Ref | 86/98952 | Ref | Ref | Ref |
|  | 0.1-0.4 servings/week | 209/101816 | 0.98 (0.81-1.19) | 0.98 (0.81-1.19) | 0.99 (0.81-1.20) | 90/102319 | 1.06 (0.79-1.43) | 1.14 (0.84-1.53) | 1.16 (0.86-1.57) |
|  | 0.4–0.8 servings/week | 236/103211 | 1.10 (0.91-1.33) | 1.11 (0.92-1.34) | 1.12 (0.93-1.35) | 76/103969 | 0.92 (0.68-1.25) | 1.00 (0.73-1.36) | 1.03 (0.75-1.41) |
|  | 0.8–1.7 servings/week | 239/102553 | 1.04 (0.86-1.25) | 1.05 (0.87-1.27) | 1.06 (0.88-1.28) | 101/103123 | 1.13 (0.84-1.50) | 1.27 (0.95-1.71) | 1.32 (0.98-1.77) |
|  | >1.7 servings/week | 236/103109 | 1.11 (0.92-1.34) | 1.13 (0.93-1.37) | 1.15 (0.95-1.40) | 102/103714 | 1.14 (0.85-1.52) | 1.28 (0.95-1.72) | 1.34 (0.99-1.80) |
|  | *P* _trend_ |  | 0.228 | 0.133 | 0.102 |  | 0.001 | <0.001 | <0.001 |
| Pastries | <1.3 servings/week | 154/99702 | Ref | Ref | Ref | 87/100040 | Ref | Ref | Ref |
|  | 1.3-2.8 servings/week | 208/102491 | 1.11 (0.90-1.37) | 1.11 (0.90-1.37) | 1.11 (0.90-1.37) | 73/103037 | 0.72 (0.53-0.98) | 0.76 (0.55-1.04) | 0.77 (0.56-1.06) |
|  | 2.8-4.4 servings/week | 219/103274 | 1.01 (0.82-1.25) | 1.01 (0.81-1.24) | 1.02 (0.82-1.26) | 89/103820 | 0.76 (0.56-1.02) | 0.82 (0.60-1.11) | 0.84 (0.62-1.14) |
|  | 4.4-6.7 servings/week | 288/102487 | 1.14 (0.94-1.40) | 1.12 (0.92-1.38) | 1.14 (0.93-1.40) | 97/103275 | 0.70 (0.52-0.94) | 0.78 (0.57-1.05) | 0.80 (0.59-1.09) |
|  | >6.7 servings/week | 260/101141 | 0.93 (0.75-1.14) | 0.91 (0.73-1.13) | 0.92 (0.74-1.15) | 109/101906 | 0.69 (0.51-0.93) | 0.76 (0.56-1.05) | 0.80 (0.58-1.10) |
|  | *P* _trend_ |  | 0.368 | 0.305 | 0.387 |  | 0.028 | 0.107 | 0.182 |
| Sweets | no intake | 251/100899 | Ref | Ref | Ref | 97/101589 | Ref | Ref | Ref |
|  | <0.1 servings/week | 246/99727 | 0.94 (0.78-1.12) | 0.95 (0.79-1.15) | 0.95 (0.79-1.15) | 133/100247 | 1.30 (1.00-1.69) | 1.14 (0.86-1.51) | 1.14 (0.86-1.51) |
|  | 0.1-0.4 servings/week | 243/102983 | 0.95 (0.80-1.14) | 0.95 (0.79-1.14) | 0.95 (0.79-1.14) | 85/103694 | 0.91 (0.68-1.22) | 0.85 (0.63-1.14) | 0.85 (0.63-1.15) |
|  | 0.4-1.0 servings/week | 223/102844 | 1.00 (0.83-1.19) | 1.00 (0.83-1.21) | 1.00 (0.83-1.21) | 79/103490 | 0.99 (0.73-1.33) | 0.89 (0.65-1.21) | 0.90 (0.66-1.22) |
|  | >1.0 servings/week | 166/102643 | 0.92 (0.76-1.12) | 0.94 (0.76-1.16) | 0.94 (0.77-1.16) | 61/103057 | 0.96 (0.70-1.33) | 0.82 (0.59-1.15) | 0.84 (0.60-1.18) |
|  | *P* _trend_ |  | 0.153 | 0.210 | 0.213 |  | 0.678 | 0.782 | 0.958 |
| Chocolate | <0.05 servings/week | 221/97822 | Ref | Ref | Ref | 120/98250 | Ref | Ref | Ref |
|  | 0.05-0.4 servings/week | 246/101412 | 1.00 (0.84-1.20) | 1.00 (0.83-1.20) | 1.00 (0.83-1.20) | 107/102068 | 0.85 (0.66-1.11) | 0.94 (0.72-1.22) | 0.94 (0.72-1.22) |
|  | 0.4–0.7 servings/week | 240/103202 | 0.98 (0.81-1.17) | 0.98 (0.82-1.18) | 0.99 (0.82-1.19) | 80/103902 | 0.63 (0.47-0.84) | 0.71 (0.53-0.94) | 0.72 (0.54-0.96) |
|  | 0.7–1.4 servings/week | 200/103653 | 0.82 (0.68-1.00) | 0.83 (0.68-1.01) | 0.84 (0.69-1.02) | 71/104166 | 0.56 (0.42-0.76) | 0.64 (0.47-0.87) | 0.65 (0.48-0.88) |
|  | >1.4 servings/week | 222/103007 | 0.92 (0.76-1.12) | 0.94 (0.77-1.15) | 0.96 (0.78-1.17) | 77/103691 | 0.61 (0.46-0.82) | 0.67 (0.50-0.92) | 0.68 (0.50-0.92) |
|  | *P* _trend_ |  | 0.729 | 0.952 | 0.877 |  | 0.018 | 0.043 | 0.043 |
| Sugar | <0.3 servings/week | 184/102481 | Ref | Ref | Ref | 77/103041 | Ref | Ref | Ref |
|  | 0.3-1.6 servings/week | 225/103426 | 1.01 (0.83-1.22) | 1.00 (0.83-1.22) | 1.01 (0.83-1.23) | 80/104012 | 0.88 (0.64-1.20) | 0.93 (0.68-1.27) | 0.94 (0.68-1.28) |
|  | 1.6–4.1 servings/week | 261/102642 | 1.03 (0.85-1.25) | 1.04 (0.85-1.26) | 1.04 (0.86-1.26) | 91/103405 | 0.88 (0.64-1.19) | 0.94 (0.69-1.28) | 0.96 (0.70-1.30) |
|  | 4.1–10.5 servings/week | 243/101439 | 0.94 (0.78-1.15) | 0.95 (0.78-1.16) | 0.95 (0.78-1.16) | 103/101979 | 0.93 (0.69-1.26) | 1.04 (0.76-1.41) | 1.07 (0.78-1.45) |
|  | >10.5 servings/week | 216/99108 | 1.11 (0.90-1.36) | 1.13 (0.91-1.40) | 1.13 (0.91-1.40) | 104/99641 | 1.09 (0.79-1.48) | 1.12 (0.81-1.55) | 1.13 (0.82-1.57) |
|  | *P* _trend_ |  | 0.110 | 0.062 | 0.066 |  | 0.015 | 0.114 | 0.141 |
| Jam and marmalade | <0.5 servings/week | 187/100200 | Ref | Ref | Ref | 95/100642 | Ref | Ref | Ref |
|  | 0.5-3.1 servings/week | 209/103410 | 0.94 (0.77-1.14) | 0.94 (0.77-1.15) | 0.95 (0.78-1.16) | 70/104030 | 0.69 (0.51-0.95) | 0.76 (0.56-1.04) | 0.77 (0.57-1.06) |
|  | 3.1–5.6 servings/week | 228/101594 | 0.94 (0.77-1.14) | 0.93 (0.77-1.14) | 0.94 (0.77-1.15) | 90/102193 | 0.74 (0.55-0.99) | 0.82 (0.61-1.09) | 0.84 (0.62-1.12) |
|  | 5.6–9.8 servings/week | 247/101657 | 0.91 (0.75-1.10) | 0.91 (0.74-1.10) | 0.91 (0.75-1.11) | 95/102331 | 0.71 (0.53-0.94) | 0.78 (0.58-1.04) | 0.80 (0.60-1.08) |
|  | >9.8 servings/week | 258/102235 | 0.91 (0.75-1.11) | 0.91 (0.75-1.12) | 0.92 (0.76-1.13) | 105/102882 | 0.68 (0.51-0.91) | 0.76 (0.56-1.03) | 0.79 (0.58-1.06) |
|  | *P* _trend_ |  | 0.765 | 0.820 | 0.931 |  | 0.126 | 0.338 | 0.435 |

Cox proportional hazard models were used to investigate associations between free sugar intake and Alzheimer’s disease and vascular dementia (yes/no) by 2014 (validated register data). Model 1 was adjusted for age, sex, season of dietary assessment, diet method, and energy intake. Model 2 was adjusted for age, sex, season of dietary assessment, diet method, energy intake, smoking status, educational level, leisure-time physical activity, alcohol consumption (g/day), body mass index, dietary habits (fruits, vegetables, nuts, processed meat, coffee, saturated fat, and fiber density), Social Network Index, prevalent diabetes, and anti-hypertensive medication use. Model 3 was adjusted for age, sex, season of dietary assessment, diet method, energy intake, smoking status, educational level, leisure-time physical activity, alcohol consumption (g/day), body mass index, dietary habits (fruits, vegetables, nuts, processed meat, coffee, saturated fat, and fiber density), Social Network Index, prevalent diabetes, anti-hypertensive medication use, and other sugar sources.

**Supplementary Table 3.** Associations between intake of free sugar and risk of all-cause dementia stratified by sex.

|  | **All-cause dementia (2014)** | | | **All-cause dementia (2020)** | | |
| --- | --- | --- | --- | --- | --- | --- |
|  | Cases/person-years | Model 1 | Model 2 | Cases/person-years | Model 1 | Model 2 |
| **Males** |  |  |  |  |  |  |
| <7.5 E% | 165/46188 | Ref | Ref | 277/53813 | Ref | Ref |
| 7.5–10 E% | 183/46319 | 0.92 (0.75-1.14) | 0.96 (0.77-1.19) | 304/54327 | 0.91 (0.78-1.08) | 0.95 (0.80-1.12) |
| 10–12.5 E% | 149/41345 | 0.80 (0.64-1.00) | 0.83 (0.66-1.05) | 255/48229 | 0.83 (0.70-0.99) | 0.87 (0.73-1.04) |
| 12.5–15 E% | 131/28758 | 1.03 (0.81-1.30) | 1.06 (0.83-1.36) | 202/33492 | 0.98 (0.81-1.17) | 1.01 (0.83-1.23) |
| >15 E% | 125/29523 | 0.98 (0.77-1.24) | 1.00 (0.77-1.29) | 209/34161 | 1.01 (0.85-1.22) | 1.02 (0.84-1.25) |
| *P* _trend_ |  | 0.62 | 0.52 |  | 0.72 | 0.70 |
| **Females** |  |  |  |  |  |  |
| <7.5 E% | 214/67939 | Ref | Ref | 384/82071 | Ref | Ref |
| 7.5–10 E% | 272/72006 | 1.06 (0.88-1.27) | 1.08 (0.90-1.30) | 452/87337 | 0.97 (0.85-1.11) | 0.98 (0.85-1.13) |
| 10–12.5 E% | 246/72344 | 0.87 (0.72-1.04) | 0.90 (0.74-1.09) | 441/87360 | 0.88 (0.77-1.01) | 0.90 (0.78-1.04) |
| 12.5–15 E% | 219/49897 | 1.08 (0.90-1.31) | 1.11 (0.90-1.36) | 334/60022 | 0.95 (0.82-1.10) | 0.96 (0.81-1.12) |
| >15 E% | 224/52416 | 1.09 (0.90-1.32) | 1.10 (0.89-1.37) | 366/62770 | 1.04 (0.90-1.20) | 1.03 (0.87-1.22) |
| *P* _trend_ |  | 0.42 | 0.46 |  | 0.70 | 0.79 |
| *P* _interaction_ |  | 0.90 | 0.91 |  | 0.93 | 0.93 |

Cox proportional hazard models were used to investigate associations between free sugar intake and all-cause dementia (yes/no) by 2014 (validated register data), and 2020 (validated register diagnoses until 2014, and unvalidated diagnoses between 2015-2020). Model 1 was adjusted for age, sex, season of dietary assessment, diet method, and energy intake. Model 2 was adjusted for age, sex, season of dietary assessment, diet method, energy intake, smoking status, educational level, leisure-time physical activity, alcohol consumption (g/day), body mass index, dietary habits (fruits, vegetables, nuts, processed meat, coffee, saturated fat, and fiber density), Social Network Index, prevalent diabetes, and anti-hypertensive medication use.

**Supplementary Table 4.** Interactions between the free sugar intake and *APOE ε4* in relation to all-cause dementia.

|  | **All-cause dementia (2014)** | | | **All-cause dementia (2020)** | | |
| --- | --- | --- | --- | --- | --- | --- |
|  | Cases/person-years | Model 1 | Model 2 | Cases/person-years | Model 1 | Model 2 |
| **APOE ε4*Free sugar** |  | 0.426 | 0.418 |  | 0.363 | 0.355 |
| **APOE ε4 carriers** |  |  |  |  |  |  |
| <7.5 E% | 179/31897 | Ref | Ref | 301/37581 | Ref | Ref |
| 7.5–10 E% | 227/33670 | 1.10 (0.91-1.34) | 1.11 (0.91-1.36) | 363/39976 | 1.05 (0.90-1.23) | 1.07 (0.91-1.25) |
| 10–12.5 E% | 198/33456 | 0.87 (0.71-1.07) | 0.88 (0.72-1.11) | 330/39403 | 0.88 (0.75-1.04) | 0.90 (0.76-1.06) |
| 12.5–15 E% | 185/22408 | 1.19 (0.96-1.46) | 1.19 (0.95-1.49) | 258/26262 | 1.03 (0.87-1.22) | 1.04 (0.87-1.25) |
| >15 E% | 166/23315 | 1.03 (0.83-1.28) | 1.02 (0.82-1.29) | 260/27345 | 1.01 (0.85-1.19) | 1.01 (0.83-1.21) |
| *P* _trend_ |  | 0.77 | 0.96 |  | 0.60 | 0.52 |
| **APOE ε4 non-carriers** |  |  |  |  |  |  |
| <7.5 E% | 188/78127 | Ref | Ref | 334/93531 | Ref | Ref |
| 7.5–10 E% | 214/79883 | 0.92 (0.75-1.12) | 0.97 (0.80-1.19) | 367/96016 | 0.88 (0.76-1.02) | 0.91 (0.78-1.06) |
| 10–12.5 E% | 184/76762 | 0.77 (0.62-0.94) | 0.82 (0.67-1.02) | 344/92073 | 0.82 (0.71-0.96) | 0.85 (0.73-1.00) |
| 12.5–15 E% | 154/53512 | 0.91 (0.73-1.13) | 0.97 (0.77-1.22) | 258/64053 | 0.88 (0.75-1.04) | 0.90 (0.76-1.08) |
| >15 E% | 171/55197 | 1.02 (0.82-1.25) | 1.06 (0.84-1.34) | 295/65579 | 1.03 (0.88-1.21) | 1.03 (0.86-1.22) |
| *P* _trend_ |  | 0.53 | 0.30 |  | 0.44 | 0.51 |
| *P* _interaction_ |  | 0.43 | 0.42 |  | 0.36 | 0.36 |

Cox proportional hazard models were used to investigate associations between free sugar intake and all-cause dementia (yes/no) by 2014 (validated register data), and 2020 (validated register diagnoses until 2014, and unvalidated diagnoses between 2015-2020). Model 1 was adjusted for age, sex, season of dietary assessment, diet method, and energy intake. Model 2 was adjusted for age, sex, season of dietary assessment, diet method, energy intake, smoking status, educational level, leisure-time physical activity, alcohol consumption (g/day), body mass index, dietary habits (fruits, vegetables, nuts, processed meat, coffee, saturated fat, and fiber density), Social Network Index, prevalent diabetes, and anti-hypertensive medication use. Abbreviations: APOE, apolipoprotein E.

**Supplementary Table 5.** Sensitivity analysis excluding dementia diagnosed within 10 years after baseline

|  | **All-cause dementia (2014)** | | | **All-cause dementia (2020)** | | |
| --- | --- | --- | --- | --- | --- | --- |
|  | Cases/person-years | Model 1 | Model 2 | Cases/person-years | Model 1 | Model 2 |
| **Free sugar intake** |  |  |  |  |  |  |
| <7.5 E% | 316/113704 | Ref | Ref | 598/135461 | Ref | Ref |
| 7.5–10 E% | 370/117754 | 0.97 (0.83-1.13) | 1.00 (0.86-1.17) | 671/141093 | 0.93 (0.83-1.03) | 0.95 (0.85-1.06) |
| 10–12.5 E% | 318/113154 | 0.80 (0.69-0.94) | 0.83 (0.70-0.98) | 619/135053 | 0.85 (0.75-0.95) | 0.87 (0.77-0.98) |
| 12.5–15 E% | 290/78235 | 1.05 (0.89-1.24) | 1.07 (0.90-1.27) | 476/93094 | 0.94 (0.83-1.07) | 0.96 (0.84-1.09) |
| >15 E% | 288/81517 | 1.03 (0.88-1.22) | 1.04 (0.87-1.25) | 514/96508 | 1.02 (0.90-1.15) | 1.02 (0.89-1.17) |
| *P* _trend_ |  | 0.40 | 0.43 |  | 0.71 | 0.77 |

Cox proportional hazard models were used to investigate associations between free sugar intake and all-cause dementia (yes/no) by 2014 (validated register data), and 2020 (validated register diagnoses until 2014, and unvalidated diagnoses between 2015-2020). Model 1 was adjusted for age, sex, season of dietary assessment, diet method, and energy intake. Model 2 was adjusted for age, sex, season of dietary assessment, diet method, energy intake, smoking status, educational level, leisure-time physical activity, alcohol consumption (g/day), body mass index, dietary habits (fruits, vegetables, nuts, processed meat, coffee, saturated fat, and fiber density), Social Network Index, prevalent diabetes, and anti-hypertensive medication use.

**Supplementary Table 6.** Sensitivity analysis excluding energy misreporters and participants who had made drastic dietary changes prior to baseline examinations, after which 17,369 participants remained.

|  | **All-cause dementia (2014)** | | | **All-cause dementia (2020)** | | |
| --- | --- | --- | --- | --- | --- | --- |
|  | Cases/person-years | Model 1 | Model 2 | Cases/person-years | Model 1 | Model 2 |
| **Free sugar intake** |  |  |  |  |  |  |
| <7.5 E% | 181/60326 | Ref | Ref | 332/72015 | Ref | Ref |
| 7.5–10 E% | 292/74905 | 1.11 (0.92-1.34) | 1.13 (0.93-1.36) | 499/89669 | 1.05 (0.91-1.21) | 1.06 (0.92-1.22) |
| 10–12.5 E% | 258/75622 | 0.90 (0.74-1.09) | 0.92 (0.76-1.12) | 474/90269 | 0.93 (0.81-1.07) | 0.95 (0.82-1.09) |
| 12.5–15 E% | 229/52944 | 1.11 (0.91-1.35) | 1.15 (0.93-1.41) | 345/63069 | 0.95 (0.82-1.11) | 0.97 (0.82-1.13) |
| >15 E% | 224/53387 | 1.11 (0.91-1.36) | 1.15 (0.92-1.43) | 361/63285 | 1.05 (0.90-1.22) | 1.04 (0.88-1.23) |
| *P* _trend_ |  | 0.26 | 0.22 |  | 0.85 | 0.76 |

Cox proportional hazard models were used to investigate associations between free sugar intake and all-cause dementia (yes/no) by 2014 (validated register data), and 2020 (validated register diagnoses until 2014, and unvalidated diagnoses between 2015-2020). Model 1 was adjusted for age, sex, season of dietary assessment, diet method, and energy intake. Model 2 was adjusted for age, sex, season of dietary assessment, diet method, energy intake, smoking status, educational level, leisure-time physical activity, alcohol consumption (g/day), body mass index, dietary habits (fruits, vegetables, nuts, processed meat, coffee, saturated fat, and fiber density), Social Network Index, prevalent diabetes, and anti-hypertensive medication use.

**Supplementary Table 7**. Sensitivity analysis excluding prevalent diabetes at baseline, after which 26,552 participants remained.

|  | **All-cause dementia (2014)** | | | **All-cause dementia (2020)** | | |
| --- | --- | --- | --- | --- | --- | --- |
|  | Cases/person-years | Model 1 | Model 2 | Cases/person-years | Model 1 | Model 2 |
| **Free sugar intake** |  |  |  |  |  |  |
| <7.5 E% | 318/104002 | Ref | Ref | 578/124626 | Ref | Ref |
| 7.5–10 E% | 435/114356 | 1.06 (0.91-1.22) | 1.06 (0.91-1.23) | 721/137156 | 0.97 (0.87-1.08) | 0.97 (0.87-1.09) |
| 10–12.5 E% | 376/111030 | 0.87 (0.74-1.01) | 0.86 (0.74-1.01) | 673/132606 | 0.88 (0.79-0.98) | 0.88 (0.79-0.99) |
| 12.5–15 E% | 338/77126 | 1.10 (0.94-1.29) | 1.09 (0.92-1.28) | 517/91782 | 0.97 (0.86-1.09) | 0.97 (0.85-1.10) |
| >15 E% | 338/80501 | 1.08 (0.92-1.26) | 1.05 (0.89-1.25) | 561/95287 | 1.04 (0.93-1.17) | 1.02 (0.90-1.17) |
| *P* _trend_ |  | 0.21 | 0.44 |  | 0.46 | 0.81 |

Cox proportional hazard models were used to investigate associations between free sugar intake and all-cause dementia (yes/no) by 2014 (validated register data), and 2020 (validated register diagnoses until 2014, and unvalidated diagnoses between 2015-2020). Model 1 was adjusted for age, sex, season of dietary assessment, diet method, and energy intake. Model 2 was adjusted for age, sex, season of dietary assessment, diet method, energy intake, smoking status, educational level, leisure-time physical activity, alcohol consumption (g/day), body mass index, dietary habits (fruits, vegetables, nuts, processed meat, coffee, saturated fat, and fiber density), Social Network Index, prevalent diabetes, and anti-hypertensive medication use.


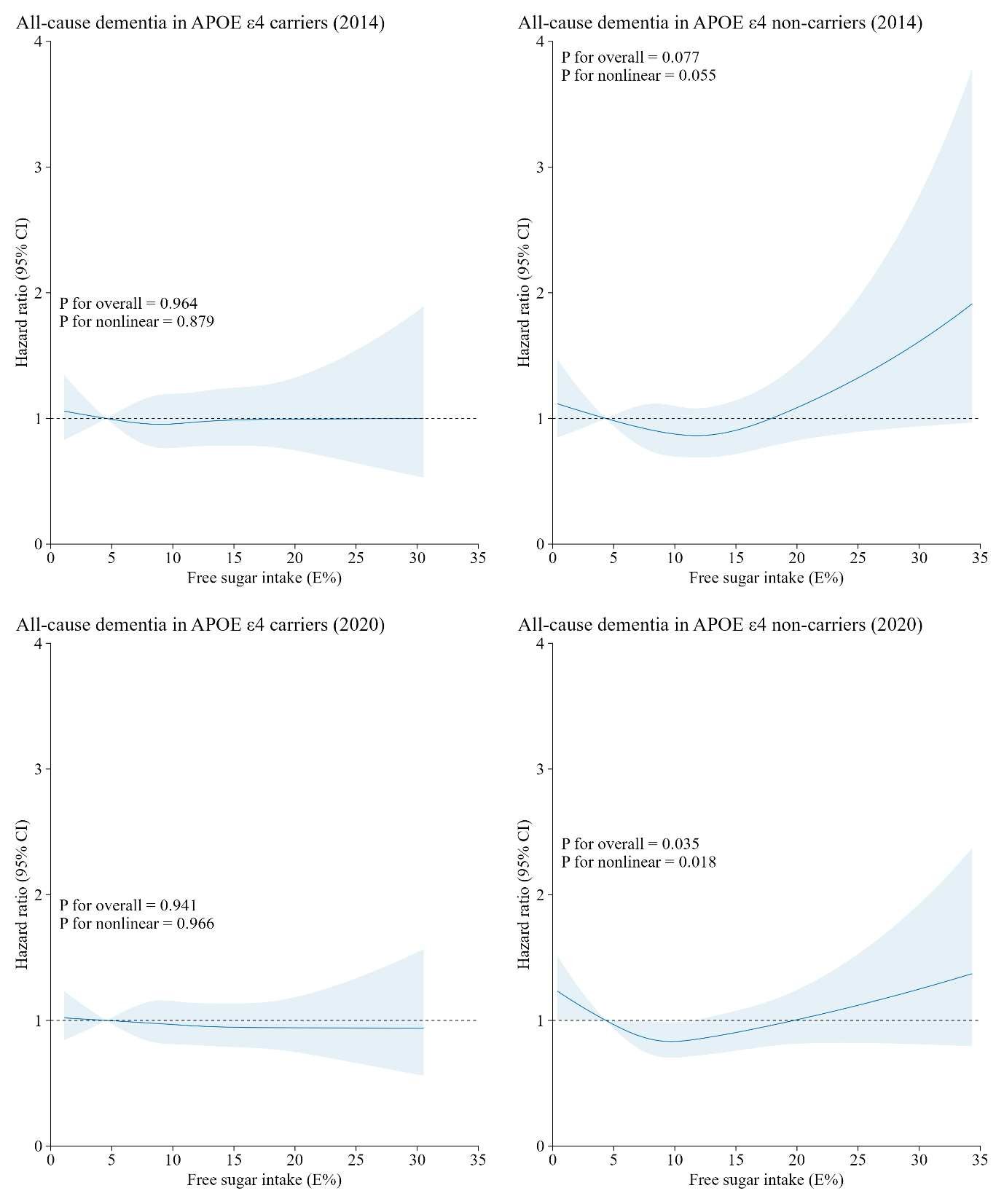
**Supplementary Figure 1.** Dose-response association between free sugar and all-cause dementia by 2014 (validated register data), and 2020 (validated register diagnoses until 2014, and unvalidated diagnoses between 2015-2020) stratified by *APOE ε4*. The model was adjusted for age, sex, season of dietary assessment, diet method, energy intake, smoking status, educational level, leisure-time physical activity, alcohol consumption, body mass index, dietary factors (fruits, vegetables, nuts, processed meat, coffee, saturated fat, and fiber density), Social Network Index, prevalent diabetes, and anti-hypertensive medication use. Intake level of 5E% was used as the reference. Abbreviations: APOE, apolipoprotein E.
